# Author Correction: Forecasting cell fate during antibiotic exposure using stochastic gene expression

**DOI:** 10.1038/s42003-019-0584-2

**Published:** 2019-08-30

**Authors:** Nicholas A. Rossi, Imane El Meouche, Mary J. Dunlop

**Affiliations:** 1 Molecular Biology, Cell Biology & Biochemistry Program, Boston University, Boston, MA 02215 USA; 2 Biological Design Center, Boston University, Boston, MA 02215 USA; 3 Department of Biomedical Engineering, Boston University, Boston, MA 02215 USA

**Keywords:** Antimicrobials, Systems biology, Information theory

Correction to: *Communications Biology* 10.1038/s42003-019-0509-0, published online 11 July 2019.

In the original version of the article, the “Methods” section contained the following two incorrect statements:

1. “All reporter plasmids have a low-copy SC101 origin of replication with a kanamycin resistance cassette and a promoter transcriptionally controlling the gene for CFP.” This has been corrected to: “All reporter plasmids have a kanamycin resistance cassette and a promoter transcriptionally controlling the gene for CFP.”
2. “the vector was originally derived from the low-copy (SC101) vector pBbS5k.” This has been corrected to: “the vector was either SC101 origin pBbS5k or ColE1 origin pBbE5k (*purA*, *inaA*, *hdeA*, σ70, *acrAB*, and *sodA* reporters use pBbS5k; all others use pBbE5k).

The errors have been corrected in the HTML and PDF versions.

