# Supplementary material for: Forecasting cell fate during antibiotic exposure using stochastic gene expression
Source: Commun Biol. 2019 Jul 11;2:259. doi: 10.1038/s42003-019-0509-0 (PMC6624276; doi:10.1038/s42003-019-0509-0)
Supplement: Supplementary file 1 — Supplementary Information [file 42003_2019_509_MOESM1_ESM.pdf]

Supplementary Information

Forecasting cell fate during antibiotic exposure using stochastic gene expression

N. A. Rossi, I. El Meouche, M. J. Dunlop

Supplementary Note

GadX controls the transcription of pH-inducible genes. Acid resistance in *E. coli* has been shown to be influenced by prior antibiotic exposure. Activation of acid response genes such as the *gadBC* operon by the antibiotic trimethoprim enables survival under subsequent acid exposure [1]. PurA plays an important role in AMP synthesis and ATP concentration has a major effect on antibiotic persistence and acid resistance [2,3]. HdeA is a periplasmic protein that supports survival in low pH [4]. In infected hosts, bacteria encounter frequent changes in environmental conditions such as fluctuations in pH, nutrients, and reactive oxygen species. Highlighting the diverse and overlapping nature of these stress response mechanisms, expression of *hdeA* and *gadX* have also been linked together under stress due to gene deletion [5].

Within the group of genes we studied are the homologous regulators Rob and SoxS and their downstream genes [6]. Rob and SoxS activate over 40 downstream genes implicated in antibiotic resistance [7]. For example, *acrA* and *acrB* are constituents of the AcrAB-TolC pump, *sodA* encodes a superoxide dismutase, and *inaA* encodes a weak acid inducible protein. OmpF is a porin involved in importing antibiotics and other compounds into the cell [8]. SoxS activates superoxide stress genes in *E. coli* such as the dismutase SodA. Oxidative stress is a major stress that bacteria encounter, both in laboratory and clinical settings. Coping with reactive oxygen species can shape bacterial response to antibiotics and have major implications on survival [9].

The transcriptional dual regulator CRP, also used in this study, is a master regulator that controls the expression of hundreds of genes. CRP is a cyclic AMP (cAMP) receptor and together they activate or repress genes involved in multiple processes such as the SOS response and antibiotic persistence [10]. Moreover, the *rrnB* P1 promoter has been shown to be a marker for bacterial persistence, responding to ATP and regulated by Fis [2].

The CodB protein is required for cytosine transport into the cell [11] and DnaQ is the epsilon unit of the DNA polymerase III, which is responsible of the proofreading capacity of the holoenzyme [12]. Induction of *dnaQ* expression occurs when the cells face mutagenic and DNA-damaging stressors [13].

References

1. Mitosch, K., Rieckh, G. & Bollenbach, T. Noisy Response to Antibiotic Stress Predicts Subsequent Single-Cell Survival in an Acidic Environment. *Cell Syst.* **4**, 393-403.e5 (2017).
2. Shan, Y. *et al.* ATP-Dependent persister formation in Escherichia coli. *MBio* **8**, e02267-16 (2017).
3. Sun, Y., Fukamachi, T., Saito, H. & Kobayashi, H. Atp requirement for acidic resistance in Escherichia coli. *J. Bacteriol.* **193**, 3072–3077 (2011).

4. Gajiwala, K. S. & Burley, S. K. HDEA, a periplasmic protein that supports acid resistance in pathogenic enteric bacteria. *J. Mol. Biol.* **295**, 605–612 (2000).
5. Wytock, T. P. *et al.* Experimental evolution of diverse Escherichia coli metabolic mutants identifies genetic loci for convergent adaptation of growth rate. *PLOS Genet.* **14**, e1007284 (2018).
6. Barbosa, T. M. & Levy, S. B. Differential expression of over 60 chromosomal genes in Escherichia coli by constitutive expression of MarA. *J. Bacteriol.* **182**, 3467–3474 (2000).
7. Martin, R. G., Bartlett, E. S., Rosner, J. L. & Wall, M. E. Activation of the Escherichia coli marA/soxS/rob Regulon in Response to Transcriptional Activator Concentration. *J. Mol. Biol.* **380**, 278–284 (2008).
8. Jaktaji, R. P. & Heidari, F. Study the Expression of ompf Gene in Esherichia coli Mutants. *Indian J. Pharm. Sci.* **75**, 540–544 (2013).
9. Van Acker, H. & Coenye, T. The Role of Reactive Oxygen Species in Antibiotic-Mediated Killing of Bacteria. *Trends in Microbiology* 456–466 (2017). doi:10.1016/j.tim.2016.12.008
10. Molina-Quiroz, R. C. *et al.* Cyclic AMP Regulates Bacterial Persistence through Repression of the Oxidative Stress Response and SOS-Dependent DNA Repair in Uropathogenic Escherichia coli. *MBio* **9**, e02144-17 (2018).
11. Danielsen, S., Kilstrup, M., Barilla, K., Jochimsen, B. & Neuhard, J. Characterization of the Escherichia coli codBA operon encoding cytosine permease and cytosine deaminase. *Mol. Microbiol.* **6**, 1335–1344 (1992).
12. Echols, H., Lu, C. & Burgers, P. M. Mutator strains of Escherichia coli, mutD and dnaQ, with defective exonucleolytic editing by DNA polymerase III holoenzyme. *Proc. Natl. Acad. Sci. U. S. A.* **80**, 2189–2192 (1983).
13. Quiñones, A. Regulation of the dnaQ gene of Escherichia coli in mutants expressing the SOS regulon constitutively. *J. Basic Microbiol.* **30**, 353–362 (1990).

Supplementary Table 1: Primers used in reporter construct design

Construct	Primers
P_{purA}	F: GGCGGTGGACTTGTG<br>R: TTTTCAAAATCACCGTTTG
P_{inaA}	F: CAATGCTTTTCAGCGTTAACTCTG<br>R: ACGACAATGACTATAGGTGGT
P_{rob}	F: CAAAATCTCAATACTTTTATTTCCG<br>R: TAATTGGATAATAGCATTTTTTGC
P_{gadX}	F: GTTGACTACCTGGGTGGTC<br>R: ACTGTTTATTAATGTAGCACGCC
P_{hdeA}	F: CCCCTGCTATCAATCTATGC<br>R: CACTGAGGTTATAACCTGGTTTTC
P_{crp}	F: ACAGAGTACGCGTACTAACCAAATC<br>R: GGGCTATCAACTGTACTGCAC
$P_{\sigma 70}$	F: AATAATTCTTGACATTTATGCTTCCG<br>R: AATTGCACGTATTATACGAGC
$P_{rrnB\ P1}$	F: CTGAACAATTATTGCCCGTTTTACAG<br>R: GAATTAAACTTCGTAATGAATTACGTGTTC
P_{acrAB}	F: GCCAGTAGATTGCACCGCG<br>R: TCGTGCTATGGTACATACATTCACA
P_{sodA}	F: GGCAATCACGGCATTAAG<br>R: TTGGTTCATTATAGTTAATTAAATG
P_{soxS}	F: CACGTTTATATCGCCGCTGATTG<br>R: TGTTGGGGAGTATAATTCCTCAAG
P_{codB}	F: GATAATTTTTCCCCCACCTTTTTGC<br>R: CCGCCGCATTCTATTCATCTG
P_{ompF}	F: CAAGTTATCTGTTTGTTAAGTCAAGCAATC<br>R: TGCAGGCATCTTTCCATTCAAAC
P_{dnaQ}	F: GTTATGGATCCACTGGGTGATAC<br>R: CGCTATTTTACGCTATCGCGG
P_{fis}	F: GCGAAGTGCGAGCAAGC<br>R: AGTTAAGAAATGACCATACTGTGACTGC
No promoter	F: GGATCCTTAACTTTAAAGAGGAGAAAG<br>R: GACCACCCAGGTAGTCAAC
gadX overexpression	F: ATGCAATCACTACATGGGAATTGT<br>R: CTATAATCTTATTCCTTCCGCAGAACG
gadX deletion	F: TGCTACATTAATAAACAGTAATATGTTTATGTA<br>ATATTAAGTCAACTATGATTCCGGGGATCCGTCGACC<br>R: CCGGGTTTTTTTATGTCTGAGTAAAACTCTA<br>TAATCTTATTCCTTCCGCTGTAGGCTGGAGCTGCTTCG

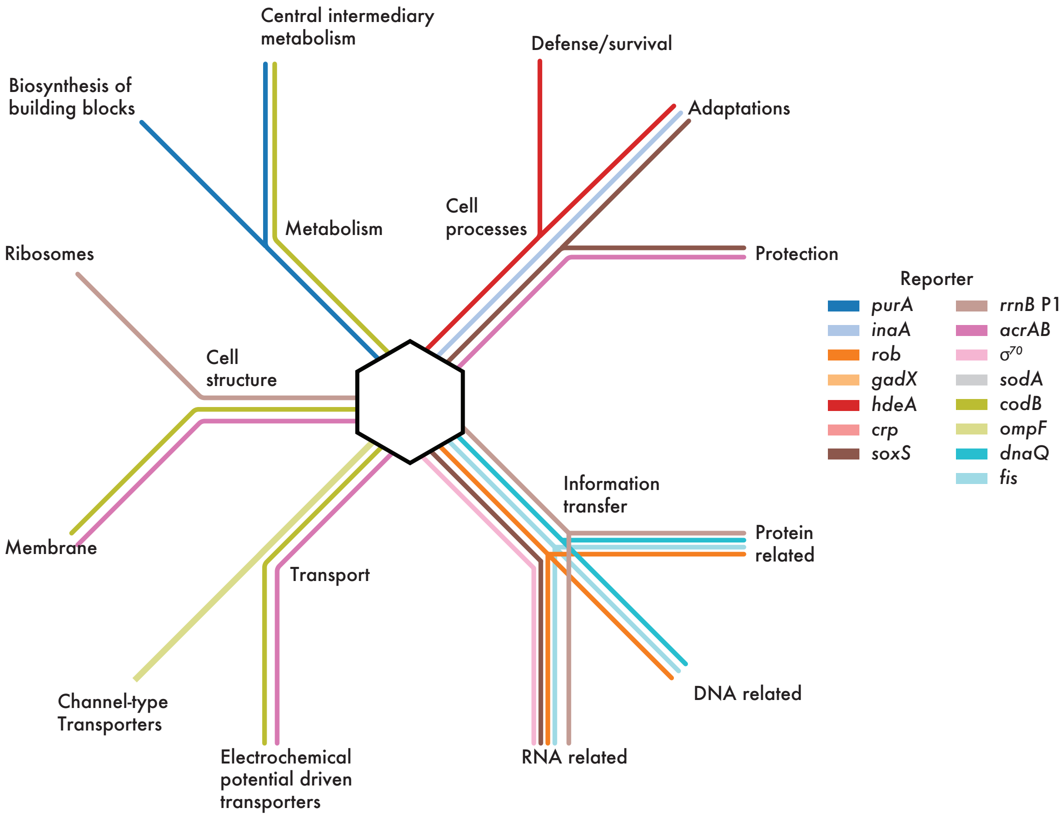

Supplementary Figure 1: Genetic ontology map for the promoters selected for study. The genetic ontology of each promoter is represented as a line connecting it through to each of its functional roles in the cell (Methods).

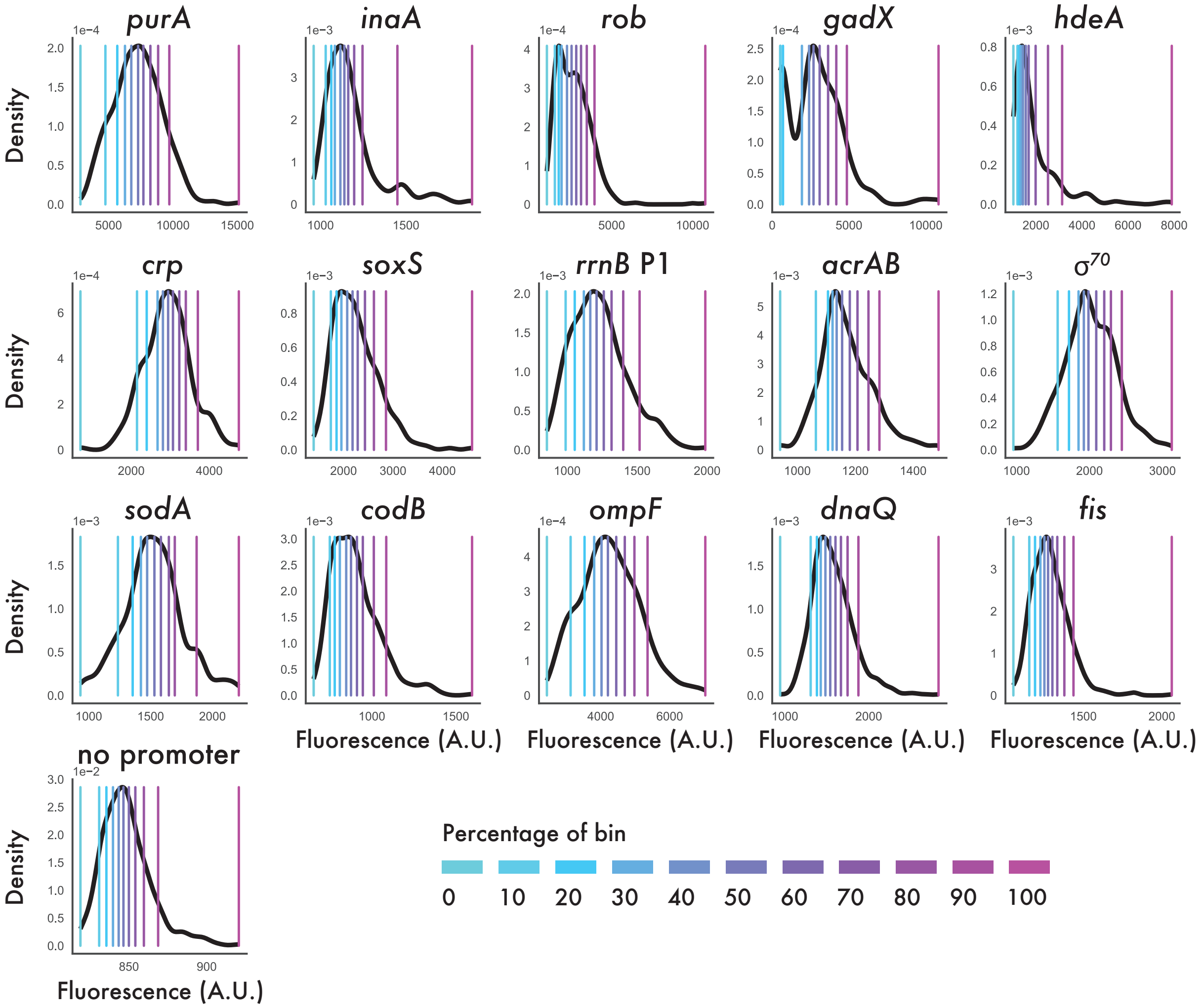

Supplementary Figure 2: Distributions and decile bins for all promoters tested.
Black lines show histograms of the distributions of initial fluorescence at t = 0 for each of the reporters. The colored lines show where each of the decile bins fall. Note that for these experiments the microscopy exposure times have been optimized to capture variation in fluorescence levels and are different for each reporter (*purA*=200ms, *inaA*=200ms, *rob*=10ms, *gadX*=10ms, *hdeA*=200ms, *crp*=10ms, *soxS*=100ms, *rrnB* P1=5ms, *acrAB*=200ms, σ^{70}=10ms, *sodA*=200ms, *codB*=10ms, *ompF*=10ms, *dnaQ*=200ms, *fis*=200ms, no promoter=200ms). Histograms with equivalent exposure times for all reporters (10ms) are shown in Supplementary Figure 3.

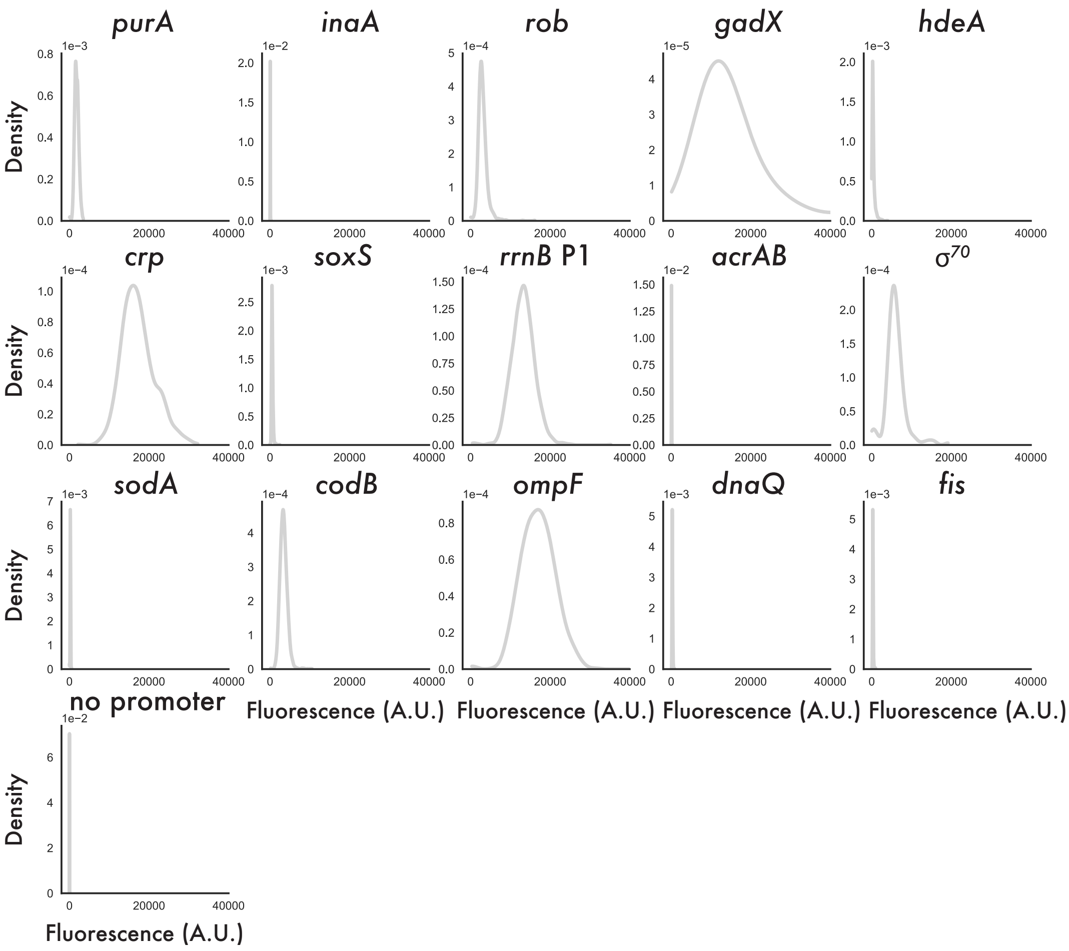

Supplementary Figure 3: Distributions using identical imaging conditions for all promoters tested.

Gray lines show histograms of the distributions of initial fluorescence at t = 0 for each of the reporters. In these experiments, identical microscopy exposure times were used for all reporters (10ms). This allows for comparison across all reporters, but results in cases where it is not possible to visualize variability well, such as for reporters where expression is low. Histograms with microscopy exposure times optimized for visualizing variability are shown in Supplementary Figure 2.

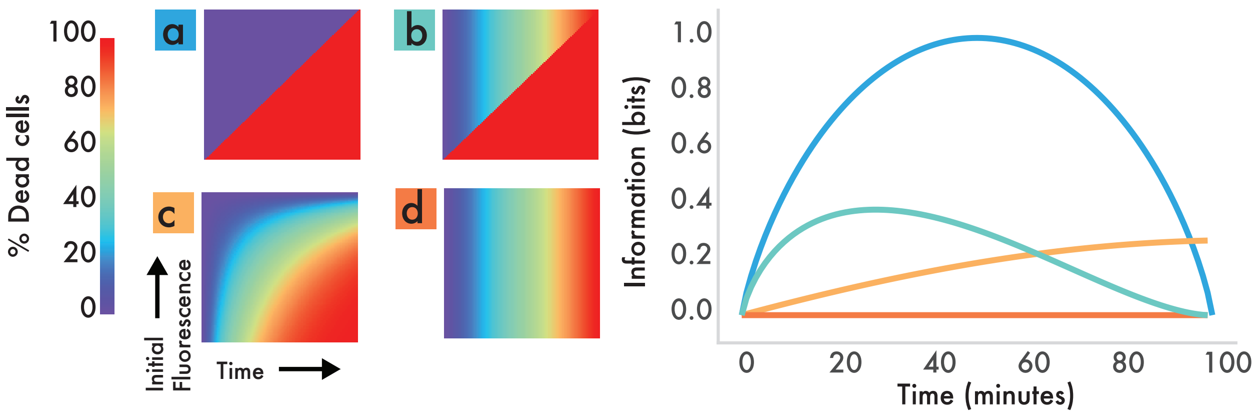

Supplementary Figure 4: Schematics of heatmaps showing variable information over time. Cartoon heatmaps of possible experimental patterns. **a** demonstrates a perfect split between alive and dead cells; the theoretical maximum information (1.0 bits) is achieved at the time of 50% cell death. **b** and **c** represent variations on this pattern. **d** demonstrates a heatmap schematic with the minimum information (0.0 bits) contained in initial fluorescence. Plots in the right panel show information over time for each of the heatmaps.

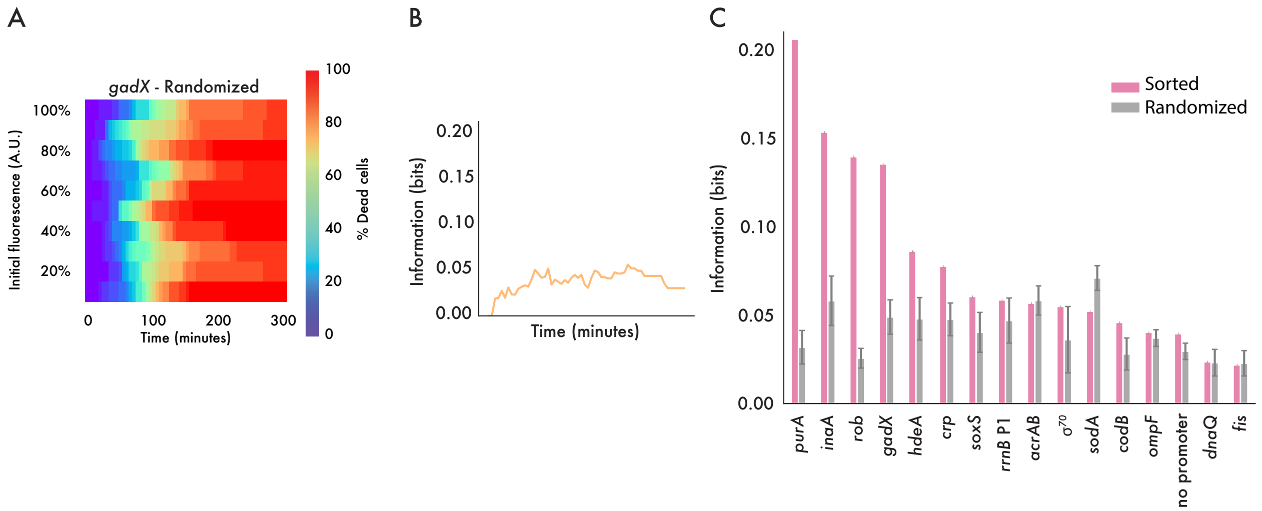

Supplementary Figure 5: Permutation test comparing data sorted by fluorescence to randomized ordering.

(A) Example of a randomized heatmap. This specific example shows P_{gadX}-*cfp* killing over time. Data are grouped into 10 bins, randomized by fluorescence value.

(B) Information over time for the randomized heatmap shown in (A).

(C) Peak mutual information values for sorted data and randomized data. The randomized data shows the mean and standard deviation for n = 100 instances of the randomization.

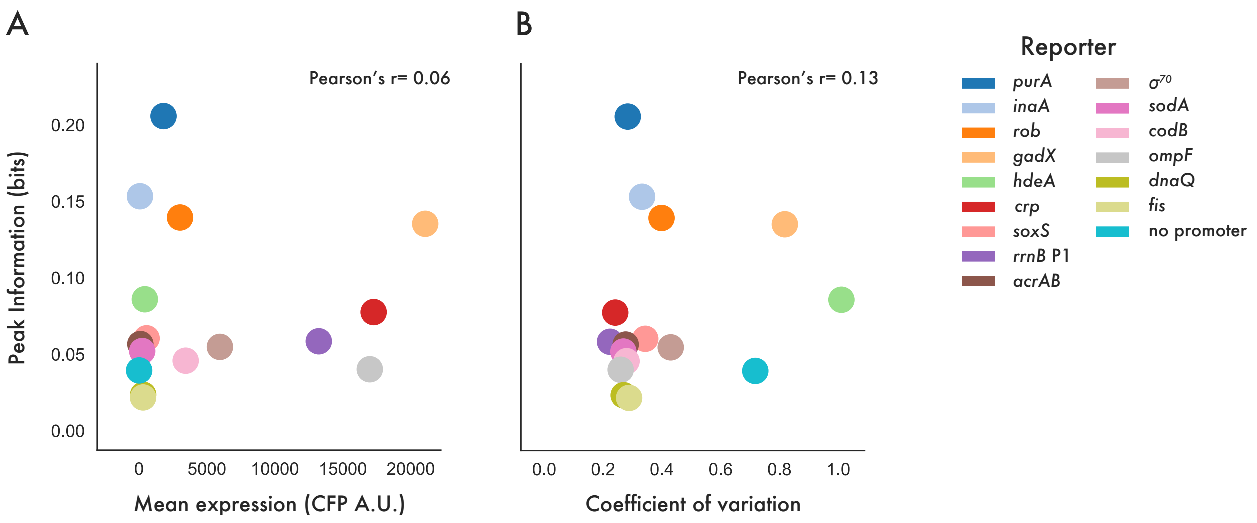

Supplementary Figure 6: Relationships between reporter statistics and information.

(A) Scatter plot for mean expression versus peak information from Fig. 2B. Pearson's r-value calculates linear correlation between the two values (1.0 = positively correlated, 0.0 = uncorrelated).

(B) Scatter plot of coefficient of variation versus peak information for each of the reporters.

Note that the plots use the mean and coefficient of variation values from the distributions with identical microscopy exposure times (Supplementary Figure 3).

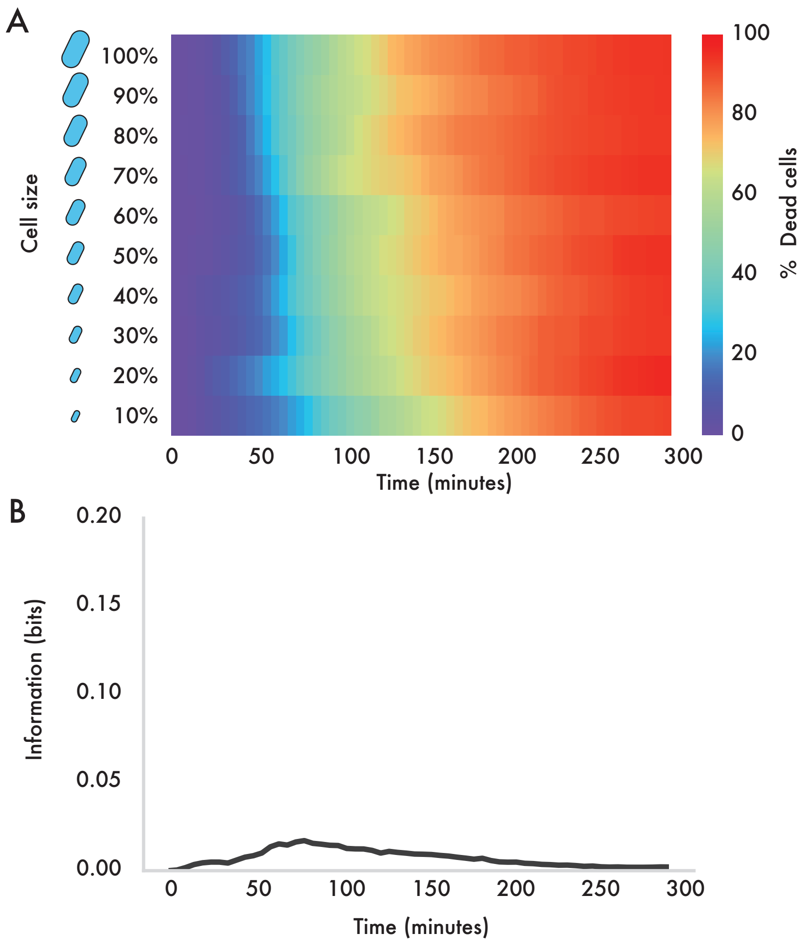

Supplementary Figure 7: Cell size as a predictor of cell fate.

(A) Heatmap that mirrors Fig. 1C but uses initial cell size instead of fluorescence on the y-axis. Data are pooled from all carbenicillin reporter experiments.

(B) Information over time for cell size. Note that the y-axis limits are the same as in Fig. 2B, allowing comparison of the peak information values.

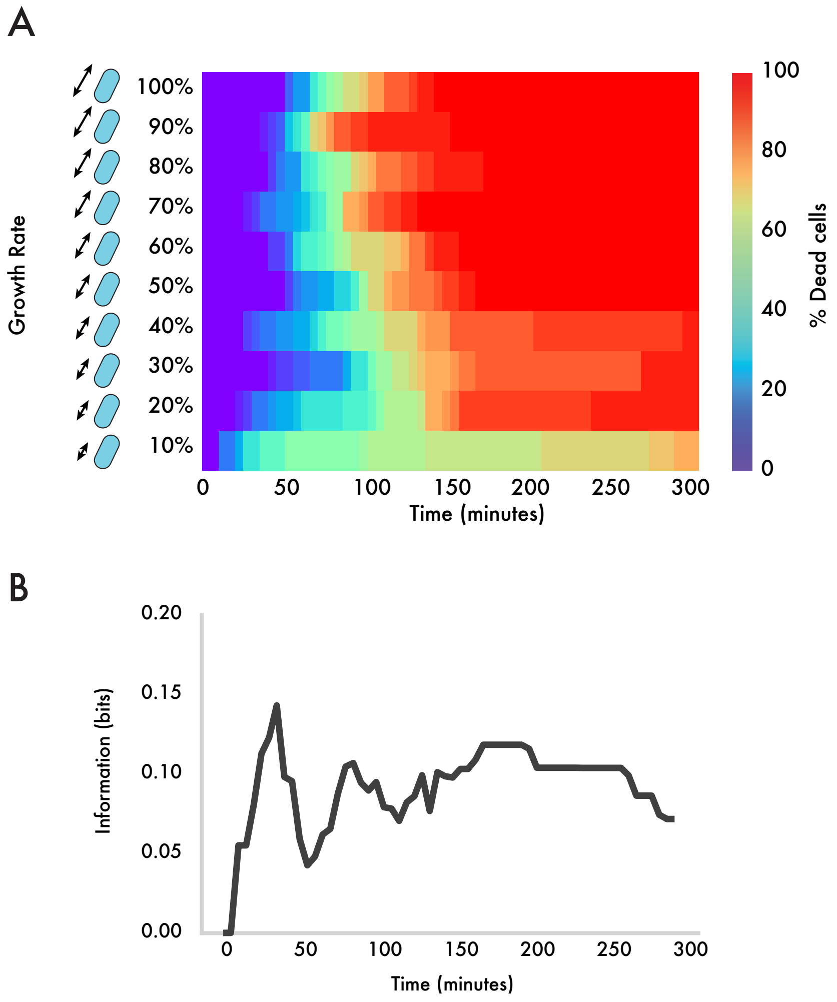

Supplementary Figure 8: Growth rate as a predictor of cell fate.

(A) Heatmap that mirrors Fig. 1C but uses initial cell growth rate instead of fluorescence on the y-axis. Data are from P_{gadX} reporter experiments with carbenicillin.

(B) Information over time for cell growth rate.

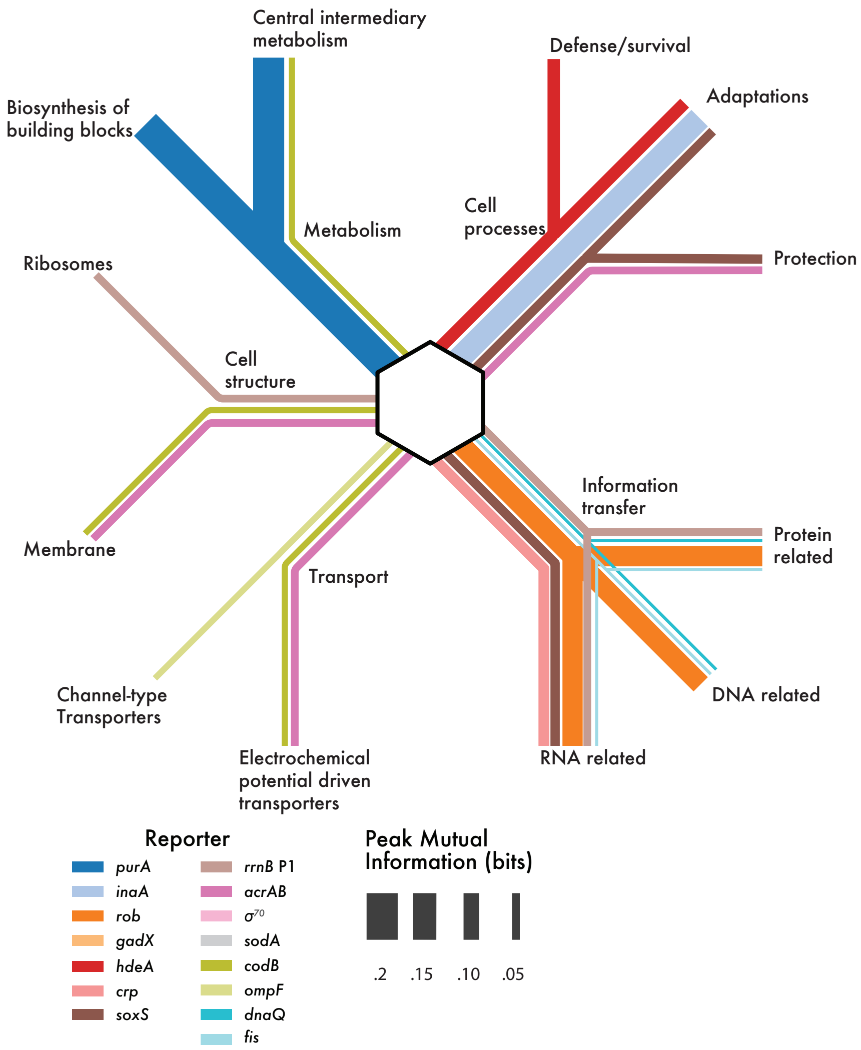

Supplementary Figure 9: Peak information from carbenicillin experiments mapped onto gene ontology.

The gene ontology map from Supplementary Figure 1 with the thickness of each connection dependent on the peak information from the carbenicillin data in Fig. 2B.

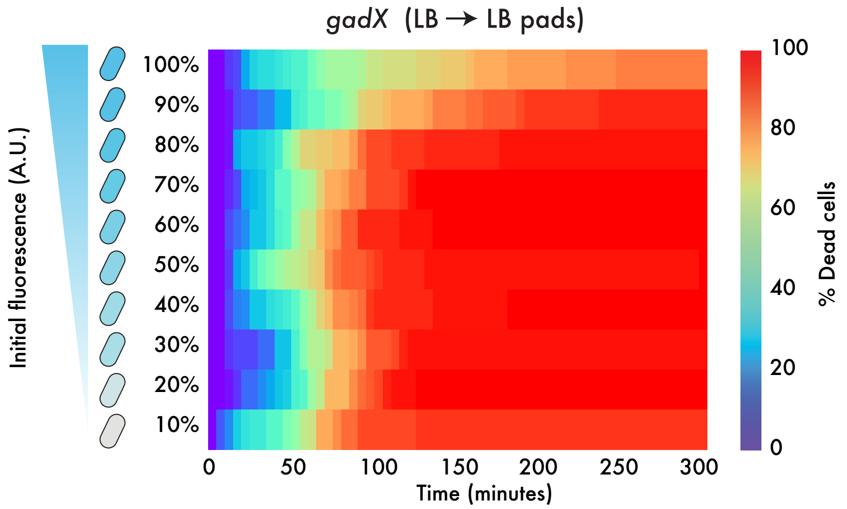

Supplementary Figure 10: Cell killing over time for cells grown on LB agarose pads. Cell killing times as a function of initial P_{gadX}-*cfp* fluorescence. In these experiments, cells were grown in LB medium and then transferred to LB agarose pads containing carbenicillin at t = 0.

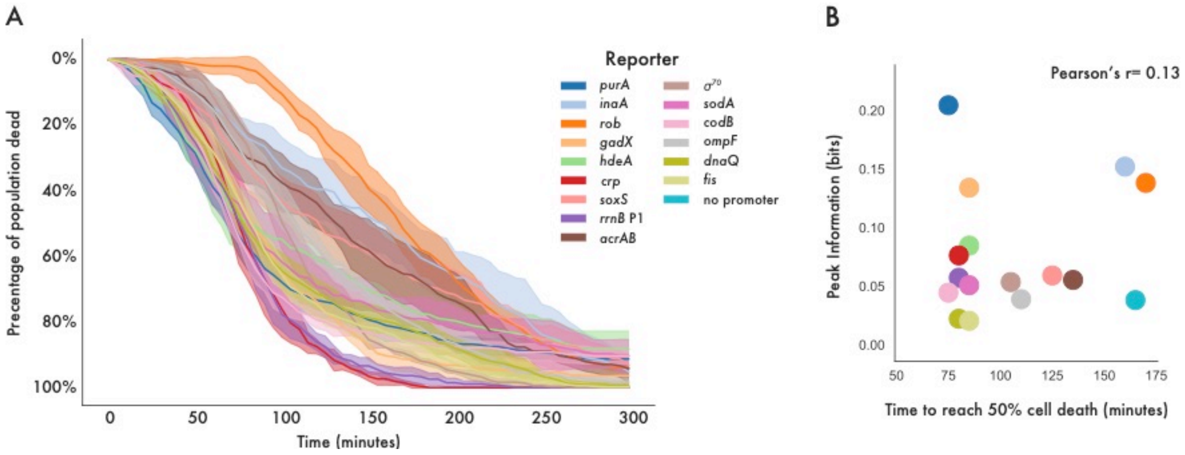

Supplementary Figure 11: Killing curves for all reporter strains over time under carbenicillin treatment.

(A) Killing curves for each strain extracted from time-lapse microscopy data. Solid lines represent the mean for at least 5 replicates while the shaded region represents the standard deviation across replicates.

(B) Scatter plot of time to reach 50% cell death versus peak mutual information. Pearson's r-value calculates linear correlation.

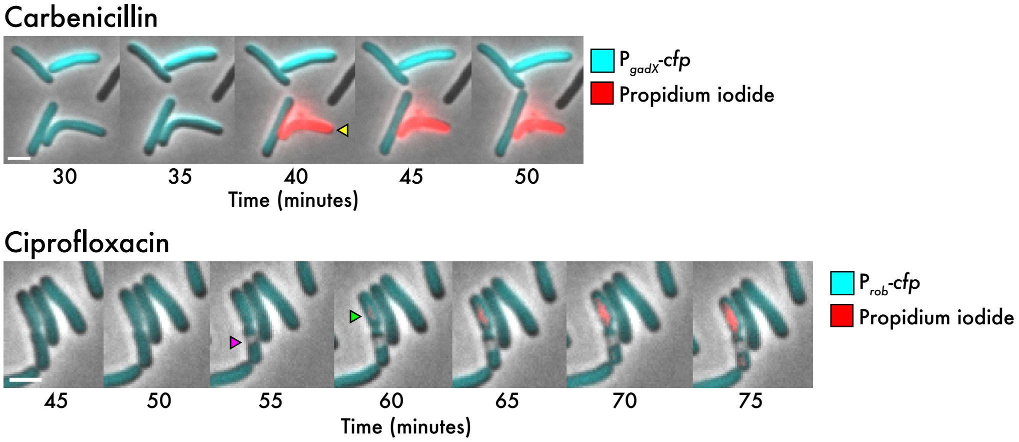

Supplementary Figure 12: Examples of identification of cell killing times.
Representative images showing how killing times were identified. Cells are marked as dead when propidium iodide stains the cell (yellow triangle in carbenicillin filmstrip, green triangle in ciprofloxacin filmstrip). Gross morphological changes are also sometimes evident and these changes are marked as cell death (magenta triangle in ciprofloxacin filmstrip). Scale bar, 2μm.

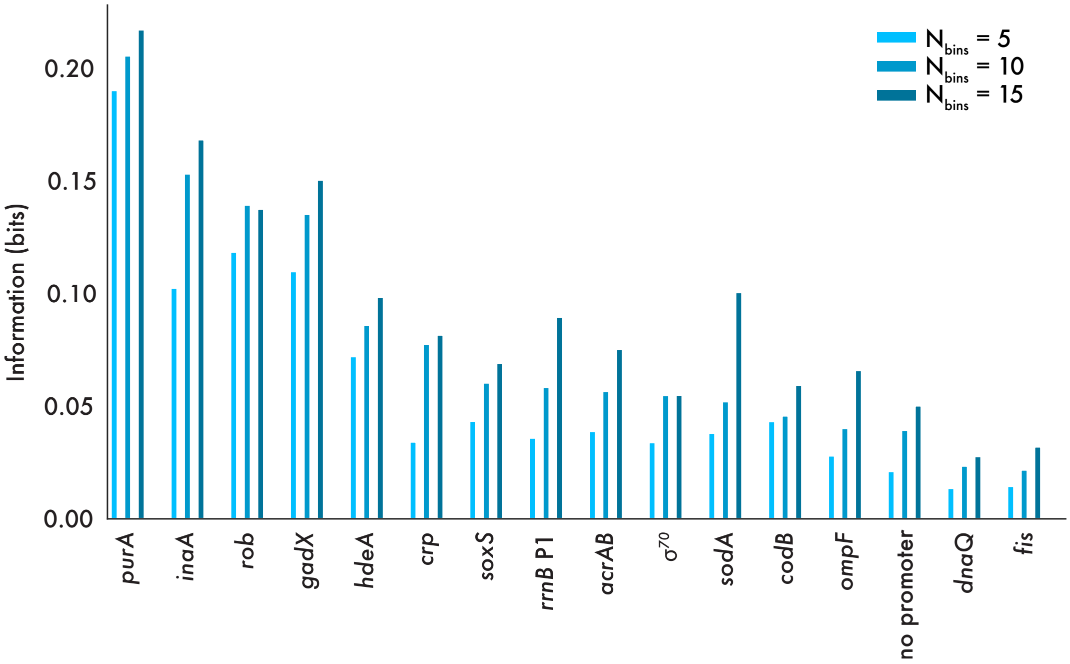

Supplementary Figure 13: Comparison of number of bins used in information calculation. Peak mutual information values for data grouped into N_{bins} = 5, 10, 15. Data sets in the main text use N_{bins} = 10.
