# Supplementary material for: Forecasting cell fate during antibiotic exposure using stochastic gene expression
Source: Commun Biol. 2019 Jul 11;2:259. doi: 10.1038/s42003-019-0509-0 (PMC6624276; doi:10.1038/s42003-019-0509-0)
Supplement: Supplementary file 2 — Description of Additional Supplementary Files [file 42003_2019_509_MOESM2_ESM.pdf]

**Supplementary Movie 1: Single-cell death under carbenicillin exposure for $P_{gadX}$-*cfp*.**

(A) Cell death over time. Red dot indicates particular point in time.

(B) Example microscopy movie of cells expressing $P_{gadX}$-*cfp* reporter (cyan) under carbenicillin exposure. Cell death is indicated by propidium iodide staining (red).

(C) Heatmap of cell death over time as a function of fluorescence at t = 0. White bar indicates particular point in time.

(D) Image showing fluorescence of individual cells at t = 0. As cells die they are eliminated from the image. Note that brighter cells tend to survive longer than dim cells.
